# Supplementary material for: Activation of the Renin–Angiotensin System Disrupts the Cytoskeletal Architecture of Human Urine-Derived Podocytes
Source: Cells. 2022 Mar 24;11(7):1095. doi: 10.3390/cells11071095 (PMC8997628; doi:10.3390/cells11071095)
Supplement: Supplementary file 1 [file cells-11-01095-s001.zip › cells-1622467-supplementary/supplement Erichsen et al.pdf]

Supplemental Material Table of Contents

Table S1: RT-qPCR Primers

Table S2: bisulfite sequencing primers

Table S3: antibodies

Table S4 UdRPC vs podocyte

Table S5 Podocyte up

Table S6 Podocyte down

Table S7 Podocyte vs 24h angII

Table S8 24h angII up

Table S9 24h angII down

Table S10 differentially expressed genes podocyte and udrpcs

Table S11 differentially expressed genes podocytes and podocytes treated with angII

Supplementary figure S1: Retinoic acid enhances the maturation of UdRPC-derived podocytes.

Supplementary figure S2: iPS cell–derived podocytes, kidney biopsy isolated human glomeruli, and mouse podocytes and Solute Carrier (SLC) Family members, reveals distinct expression patterns of UdRPCs and their differentiated counterparts.

Supplementary figure S3: Dynamic changes in the morphology of human urine derived podocytes after treatment with Angiotensin II (ANGII) after 6h.

Supplementary figure S4: DNA Methylation changes at the NPHS1 promoter upon 6h of ANGII treatment.

Supplementary figure S5: Dynamic changes in the morphology of human urine derived podocytes post treatment with Angiotensin II (ANGII) after 6h and 24h and is rescued by Losartan

Supplementary figure S6: Kegg-pathway Renin-Angiotensin system.

Supplementary figure S7: Secretome membranes and analysis of podocytes with and without 6h and 24h of Angiotensin II treatment.

Supplementary figure S8: Full size Western blot figures for A = GAPDH, B = NPHS1 and C = SYNPO.

Supplementary figure S9: Full size Western blot figures for A = GAPDH, B = NPHS1 and C = SYNPO.

Supplementary figure S10: Supplementary figure 10: Albumin uptake assay in UM51 podocytes

Supplementary figure S11: Principal component analysis of podocytes UM48 and UM51 with and without 6h and 24h of ANGII treatment

Table S1: RT-qPCR Primers

Primer name	Sequence	Annealing temperature (°C)	Product length (bp)
AGTR1 s	5'- tct cag cat tga tcg ata cc -3'	60	80
AGTR1 as	5'- tga ctt tgg cta caa gca tt -3'		
AGTR2 s	5'- tat ggc ctg ttt gtc ctc at -3'	60	114
AGTR2 as	5'- cat tgg gca tat ttc tca gg -3'		
LMX1b s	5'- cat cct cac cac gca gca g -3'	60	155
LMX1b as	5'- tct tca tct ttg ctc ttt ggt tct -3'		
NPHS1 s	5'- gcg ggt tct gct acg atg gtg -3'	60	295
NPHS1 as	5'- caa aca cac cag cct cac ccg -3'		
RPL0 s	5'- tcg aca atg gca gca tct ac -3'	60	195
RPL0 as	5'- atc cgt ctc cac aga caa gg -3'		
SYNPO s	5-' ccc caa cct ctc ctc taa cc -3'	60	116
SYNPO as	5'- atg aca cag gag gca gaa gaa t -3'		
WT1 s	5'- cac agc aca ggg tac gag a -3'	60	133
WT1 as	5'- caa gag tcg ggg cta ctc c -3'		

Table S2: bisulfite sequencing primers

Primer name	Sequence	Annealing temperature (°C)	Product length (bp)
NPHS2 bseq s	5'- gtt ttg agg atg gag agg agg -3'	55	415
NPHS2 bseq as	5'- ttc cta aaa acc taa aca tcc aac -3'		
WT1 bseq s	5'- ggg gga ggg ttg tgt tat at -3'	54	270
WT1 bseq as	5'- ctc ctt acc cca acta cc taa cta c -3'		

Table S3: antibodies

Antigen	Company	Dilution IF/WB
α-ACTININ	Abcam (#108198)	1:200/n.a.
GAPDH	Thermo Fisher Scientific (AM4300)	n.a./1:1000
CD2AP	Cell Signaling Technology (A599)	1:200/n.a.
NPHS1	Invitrogen (#PA5-20330)	1:200 /1:1000
NPHS2	Abcam (#50339)	1:200/n.a.
SYNPO	Thermo Fisher Scientific (PA5-56997)	1:200/1:1000
Anti-mouse HRP-labeled	Thermo Fisher Scientific #NA931	n.a./1:4000

Anti-rabbit HRP-labeled	Cell Signaling #7074S	n.a./1:1000

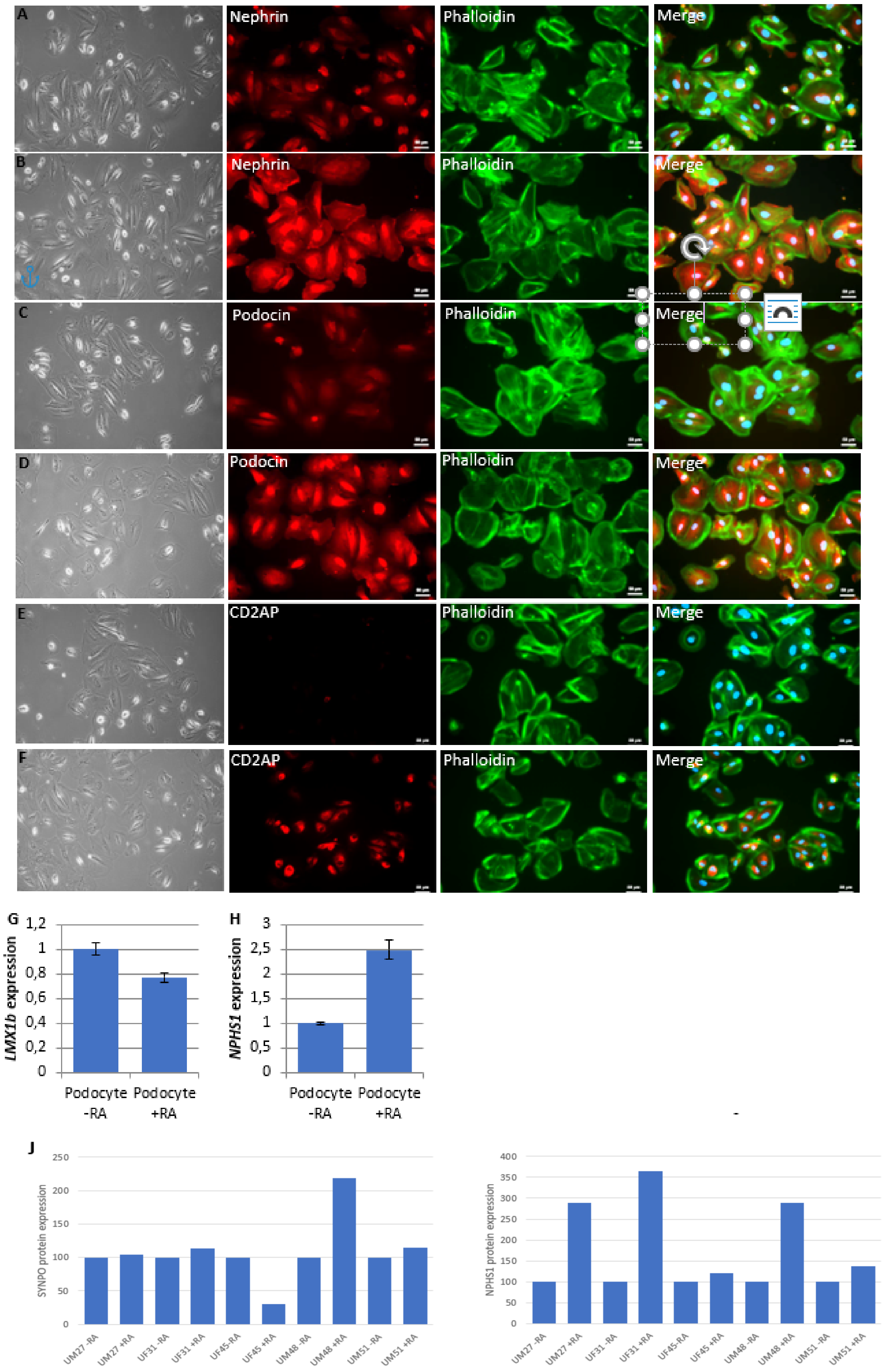

Supplementary figure S1: Retinoic acid enhances the maturation of UdRPC-derived podocytes. UdRPCs differentiated into podocytes, by low density cultivation in advanced RPMI medium supplemented and with 30 µM retinoic acid. Immunofluorescence-based detection of NPHS1, NPHS2 and CD2AP expression comparing podocytes differentiated with and without the addition of RA. Podocyte cytoskeleton was stained

with phalloidin. Expression of podocyte markers *LMX1B* and *WT1* was determined by by quantitative real time PCR (G+H). Quantification of western blot-based detection of podocyte markers Nephrin and Synaptopodin (J).

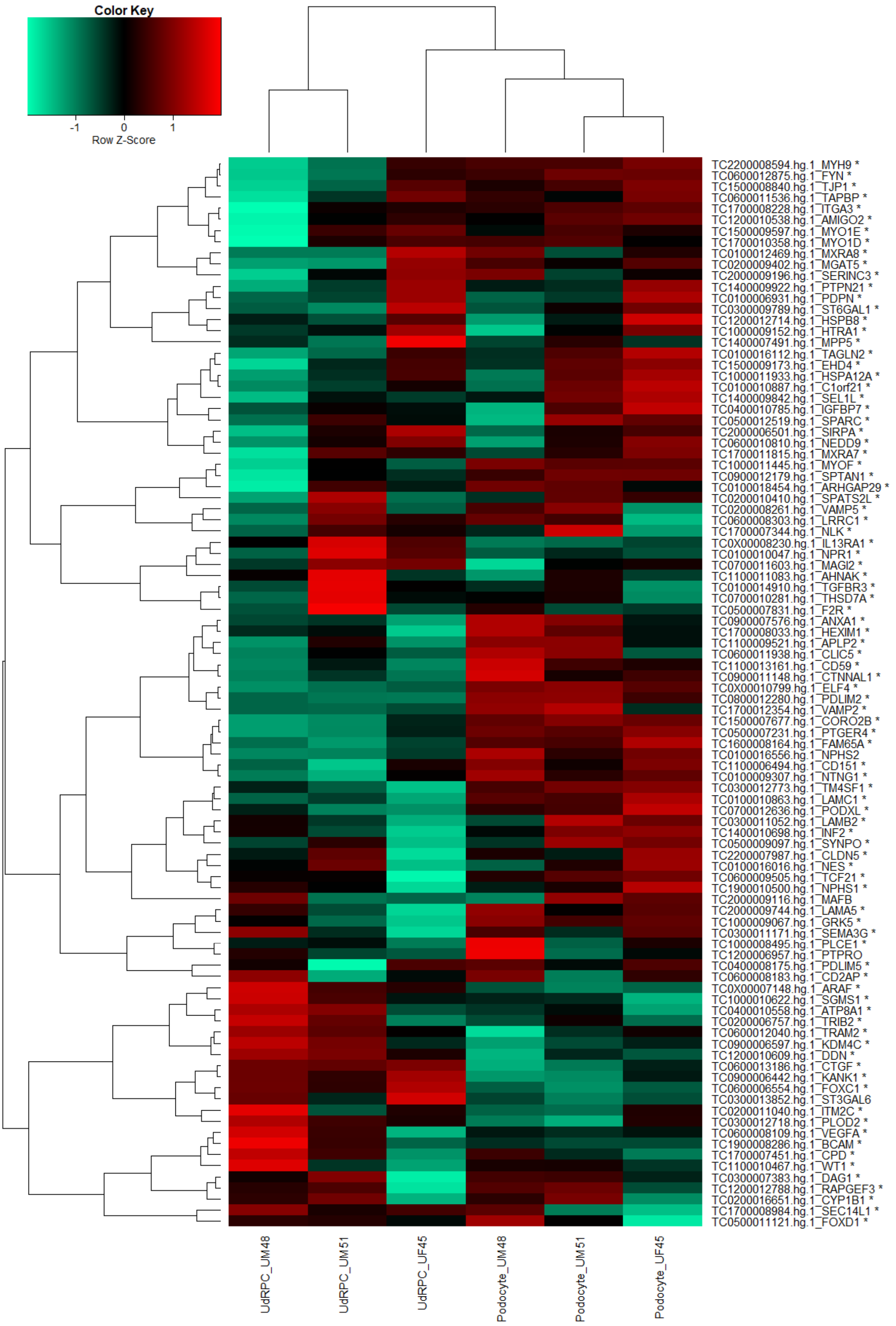

Supplementary figure S2: iPS cell–derived podocytes, kidney biopsy isolated human glomeruli, and mouse podocytes and Solute Carrier (SLC) Family members, reveals distinct expression patterns of UdRPCs and their differentiated counterparts.

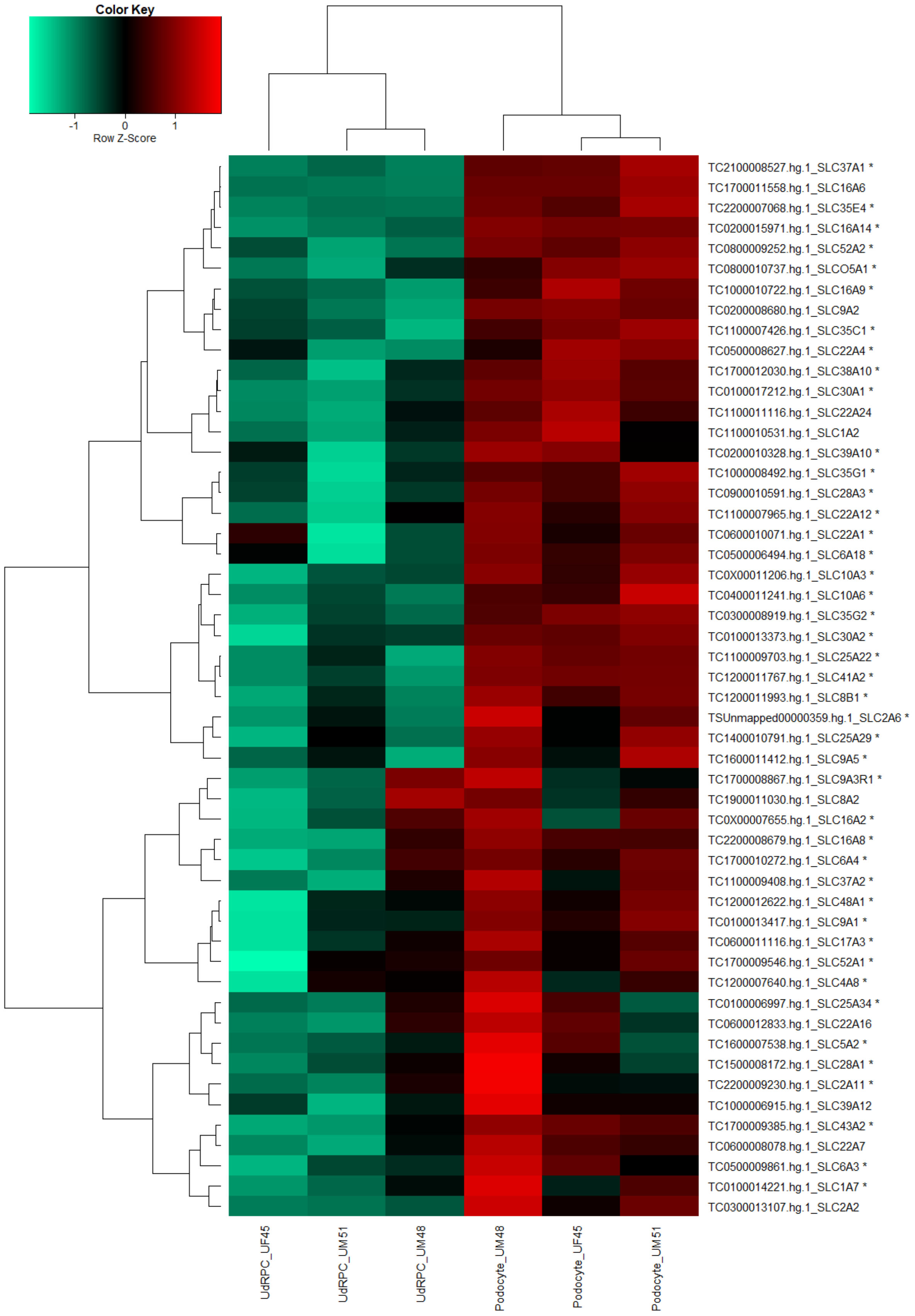

Supplementary figure S2: continuing.

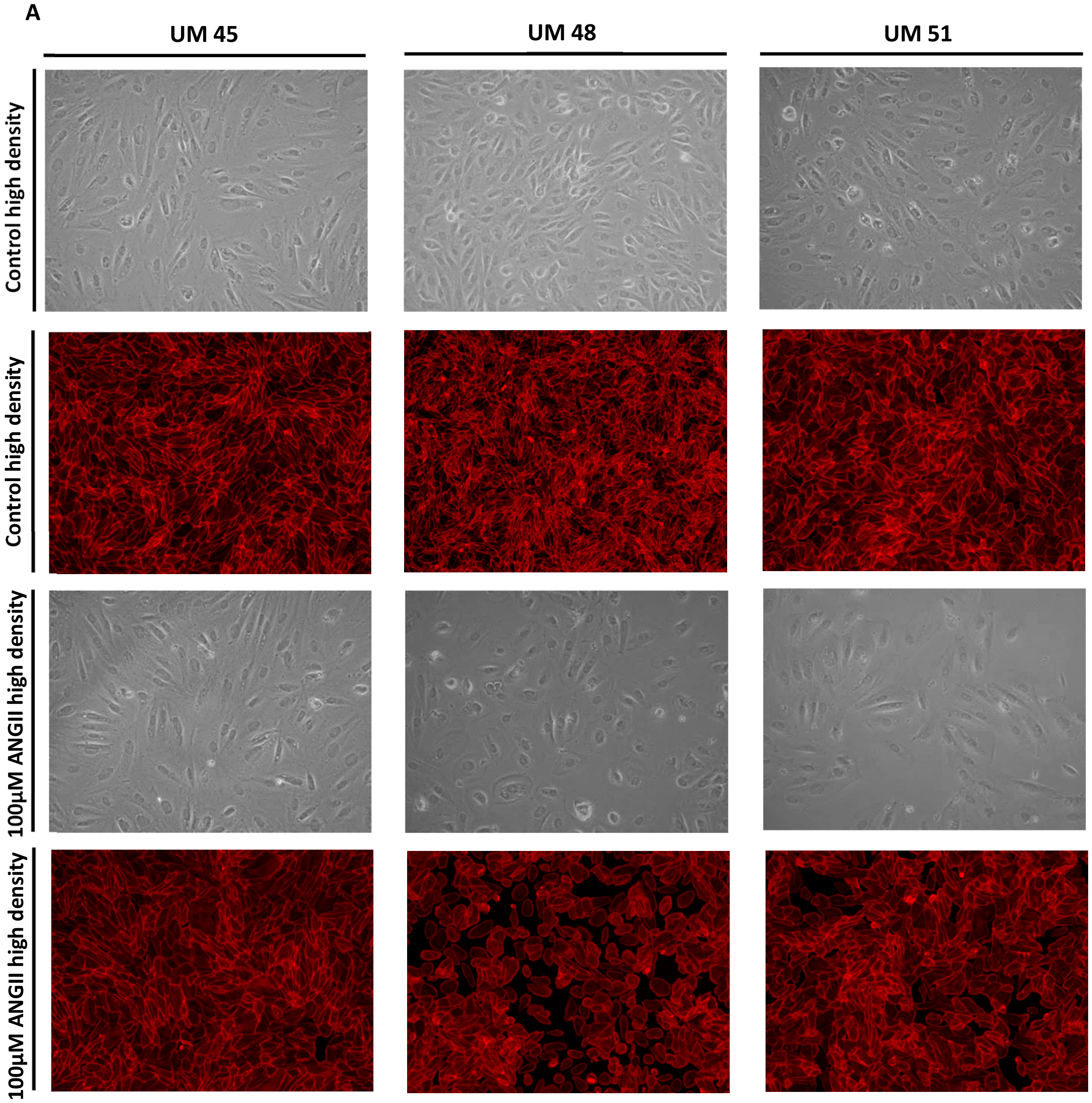

A
UM 45
UM 48
UM 51
Control high density
Control high density
100µM ANGII high density
100µM ANGII high density

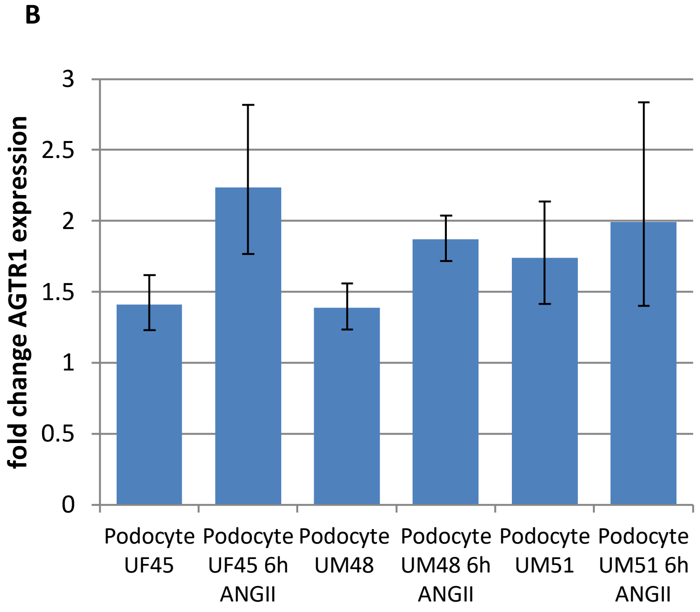

B
fold change AGTR1 expression
3
2.5
2
1.5
1
0.5
0
Podocyte UF45
Podocyte UF45 6h ANGII
Podocyte UM48
Podocyte UM48 6h ANGII
Podocyte UM51
Podocyte UM51 6h ANGII

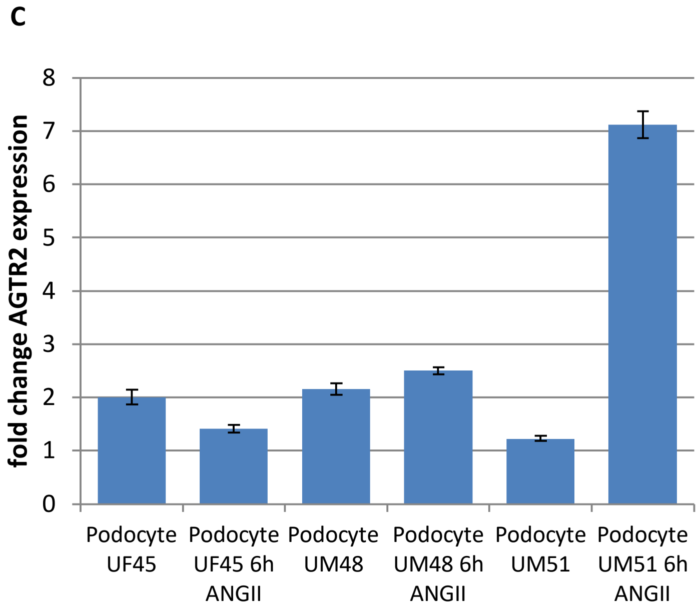

C
fold change AGTR2 expression
8
7
6
5
4
3
2
1
0
Podocyte UF45
Podocyte UF45 6h ANGII
Podocyte UM48
Podocyte UM48 6h ANGII
Podocyte UM51
Podocyte UM51 6h ANGII

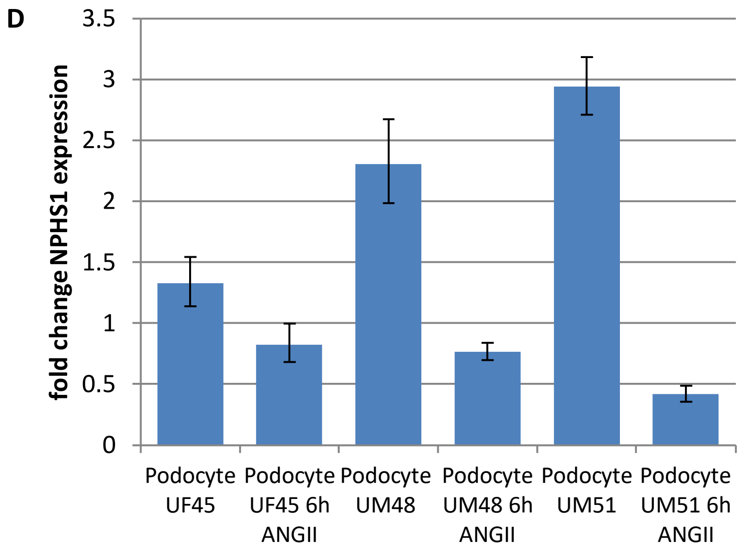

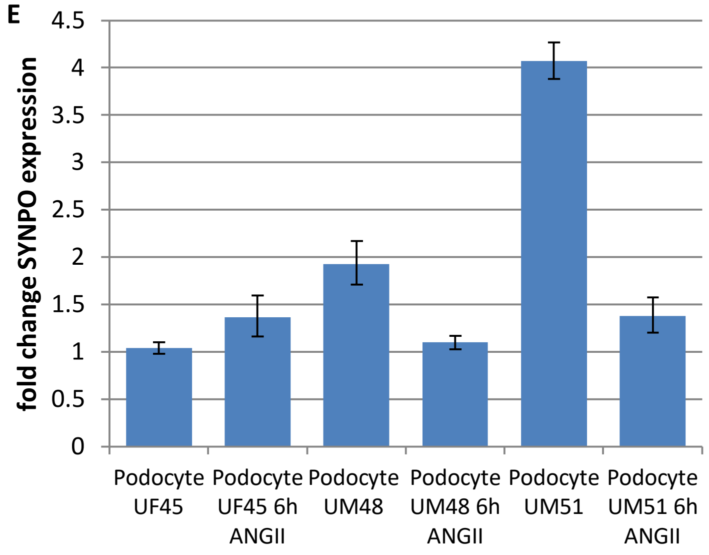

Supplementary figure S3: Dynamic changes in the morphology of human urine derived podocytes after treatment with Angiotensin II (ANGII) after 6h.

UdRPC-differentiated podocytes from UF45, UM48 and UM51. The top panel (phase contrast) show podocyte morphology. The lower two panels show morphology changes after 6h of 100 µM ANGII treatment. Podocyte cytoskeleton was visualized by immunofluorescence-based detection of α–Actinin in red (a). Expression of ANGII receptors *AGTR1* (b), *AGTR2* (c), expression podocyte markers *NPHS1* (d) and *SYNPO* (e) were determined by quantitative real time PCR normalized with the ribosomal encoding gene-RPL0.

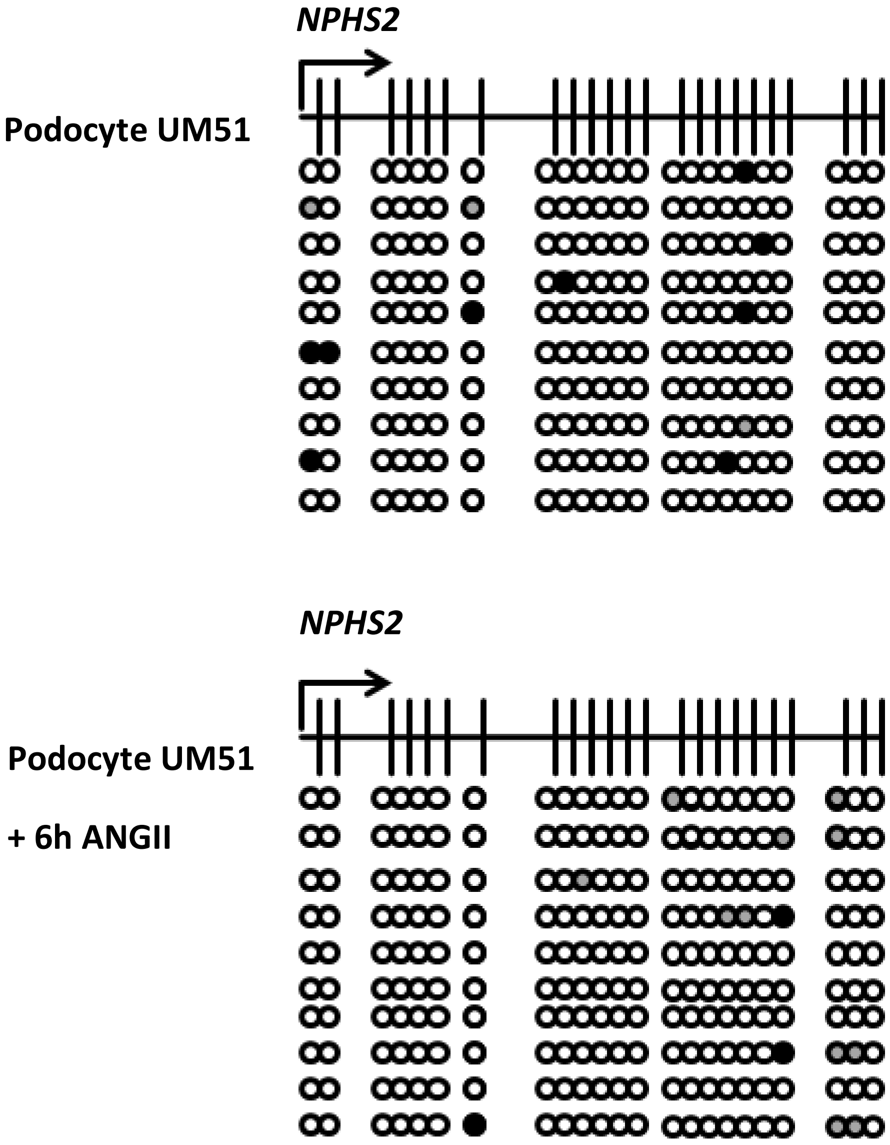

Supplementary figure S4: DNA Methylation changes at the NPHS1 promoter upon 6h of ANGII treatment.
Bisulfite genomic sequencing of a 365bp long *NPHS2* promoter fragment, spanning 23 CpG-dinucleotides, provide detailed information about the dyamic DNA Methylation changes occurring during the 6h treatment of podocytes with ANGII.

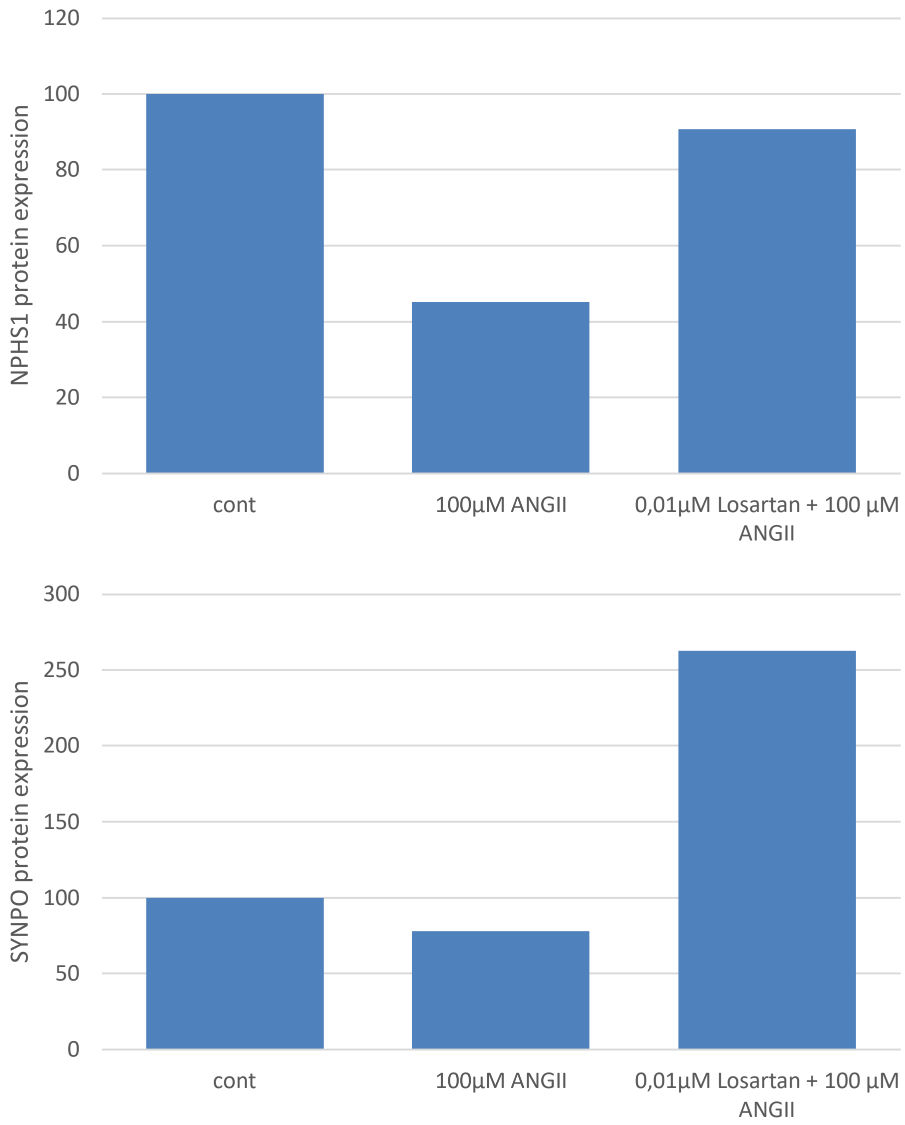

Supplementary figure S5: Dynamic changes in the morphology of human urine derived podocytes post treatment with Angiotensin II (ANGII) after 6h and 24h and is rescued by Losartan. Quantification of western blot-based detection of podocyte markers Nephrin and Synaptopodin in control podocytes compared to podocytes treated with either 100µM ANGII or a combination of 0,01µM Losartan and 100µM ANGII.

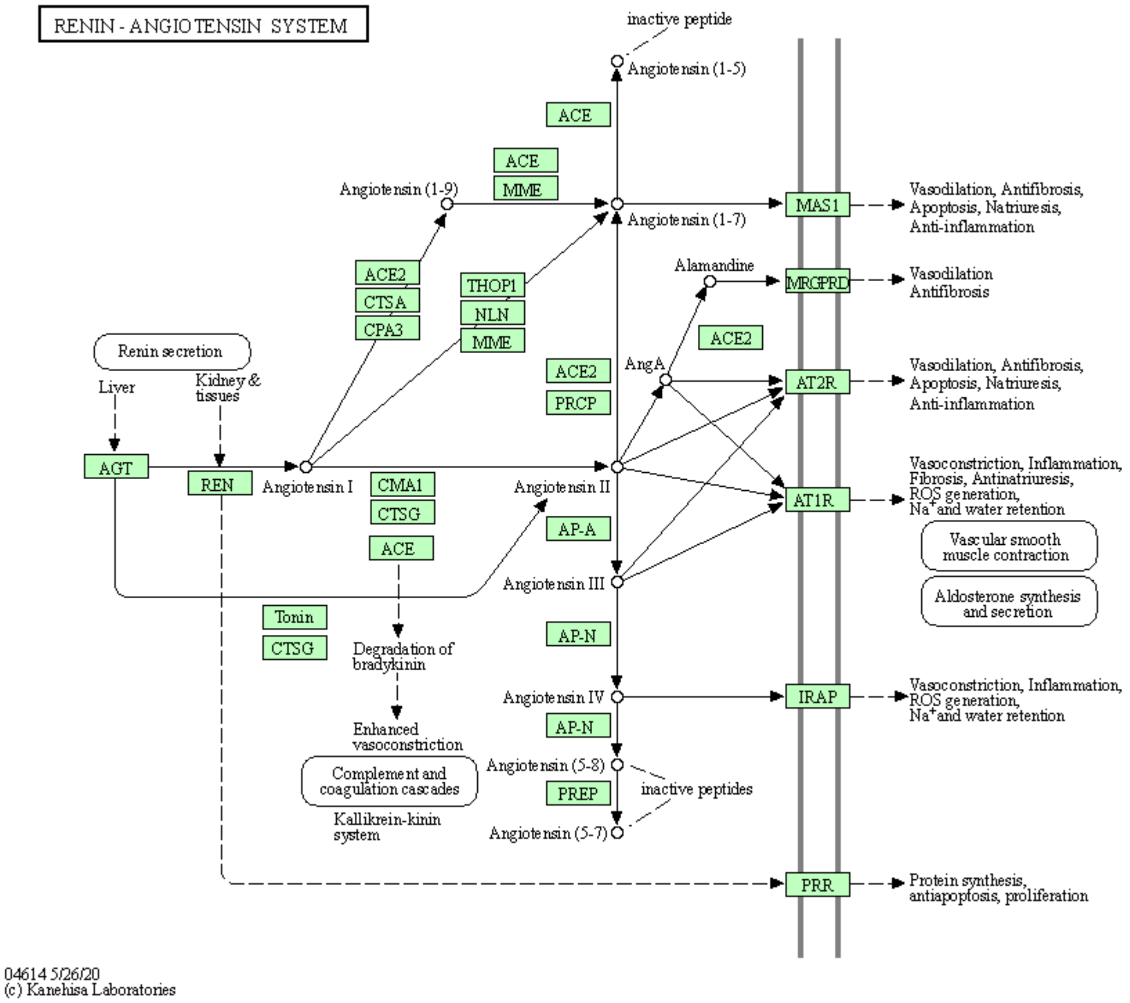

Supplementary figure S6: Kegg-pathway Renin-Angiotensin system.

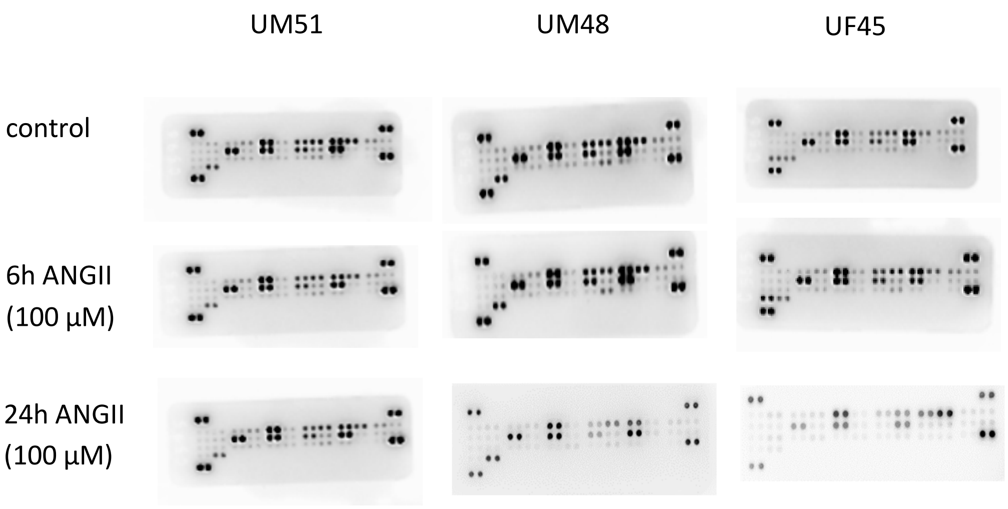

Podocytes pooled

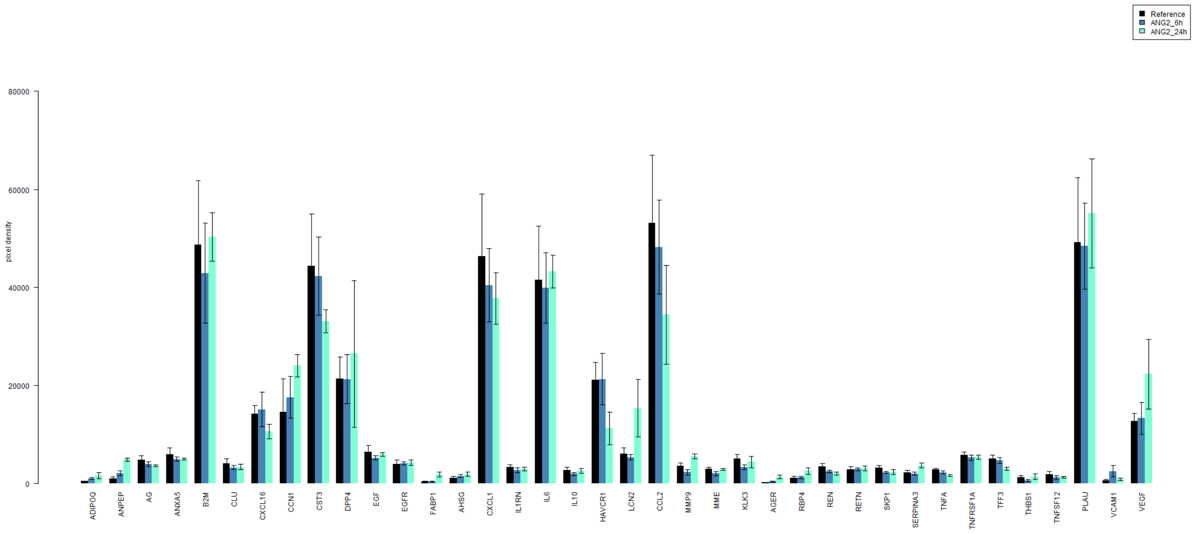

Podocyte UM48

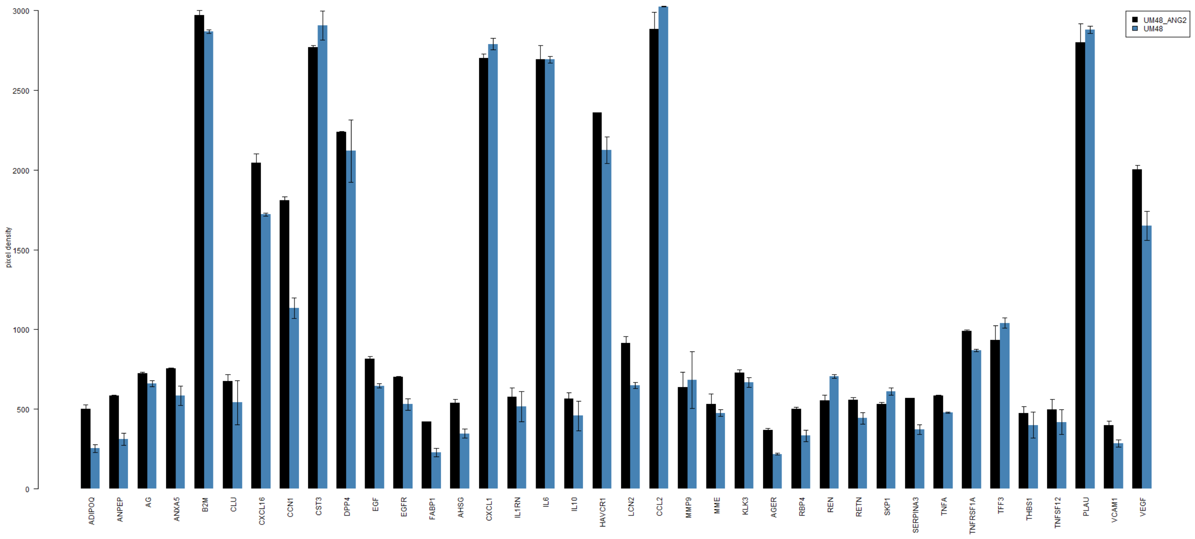

Podocyte UF45

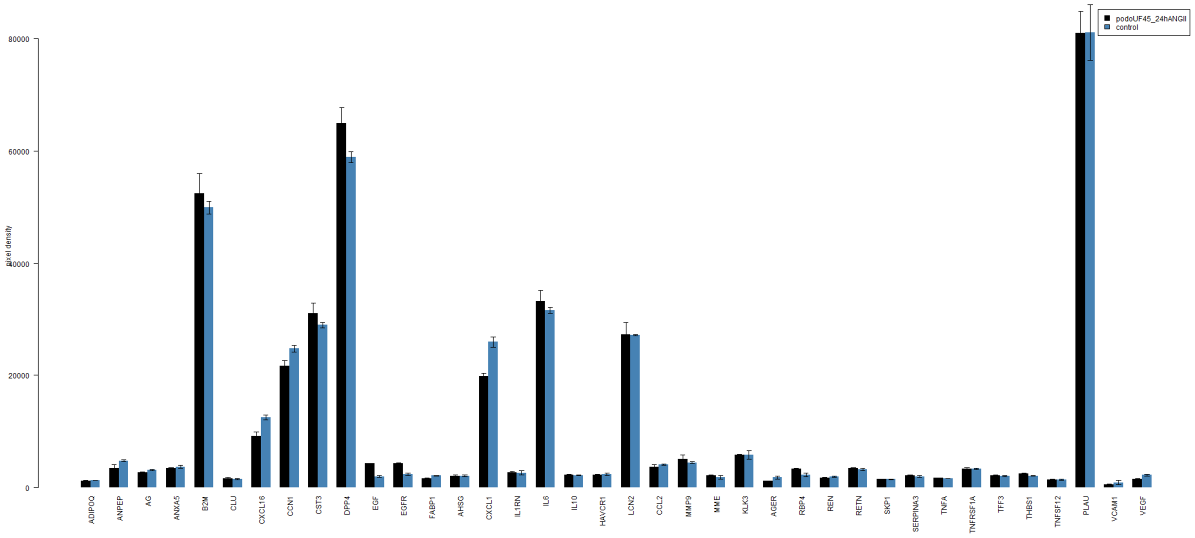

Podocyte UM51

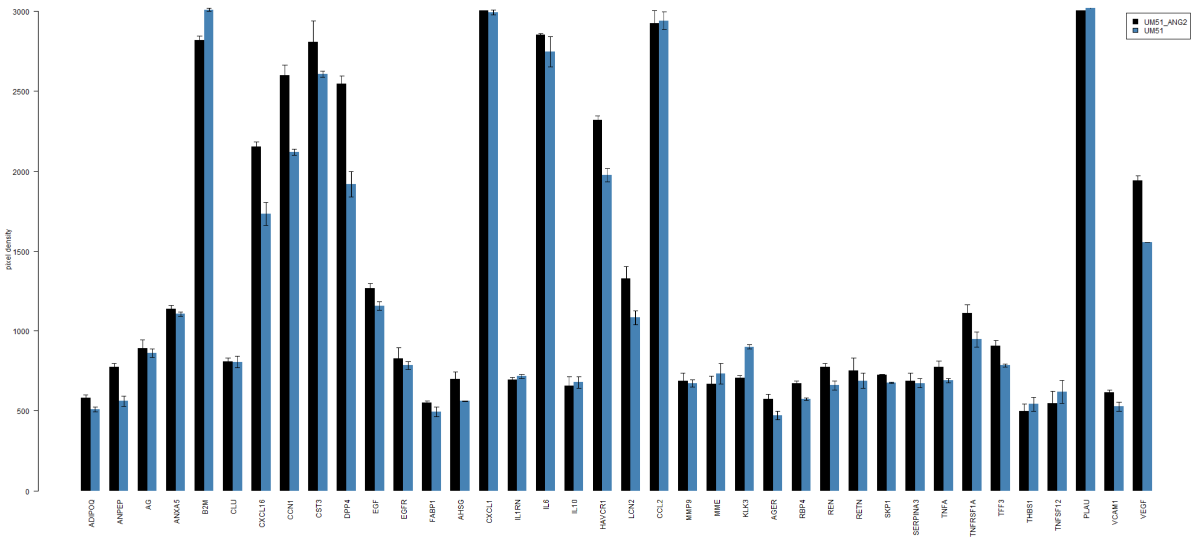

Supplementary figure S7: Secretome membranes and analysis of podocytes with and without 6h and 24h of Angiotensin II treatment.

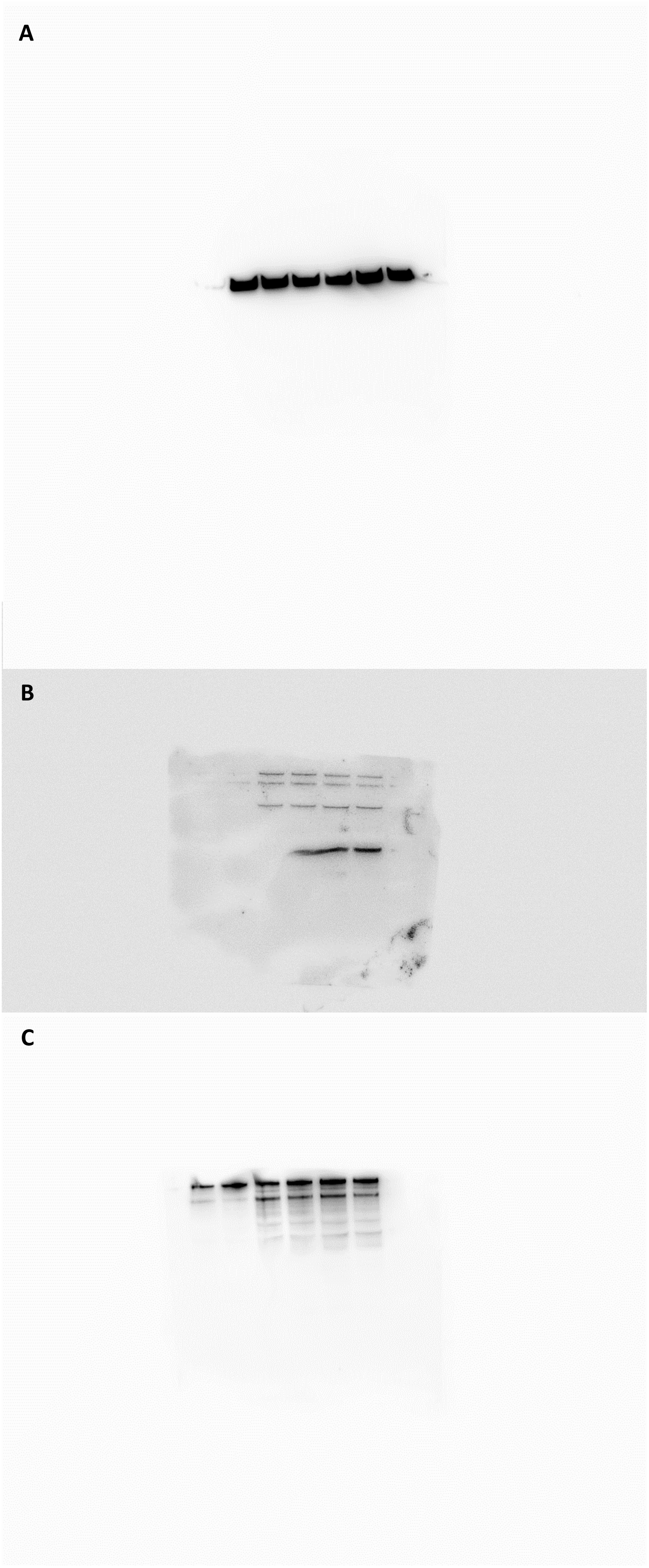

Supplementary figure S8: Full size Western blot figures for A = GAPDH, B = NPHS1 and C = SYNPO. Samples were loaded from left to right: UF45 +RA, UF45 -RA, UM51 +RA, UM51 -RA, UM27 +RA and UM27 -RA.

A

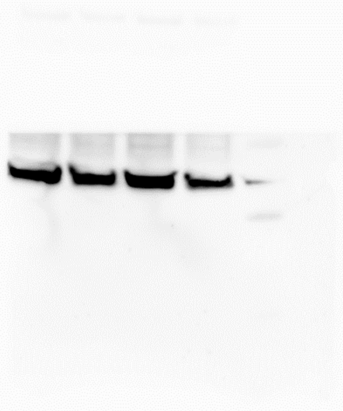

B

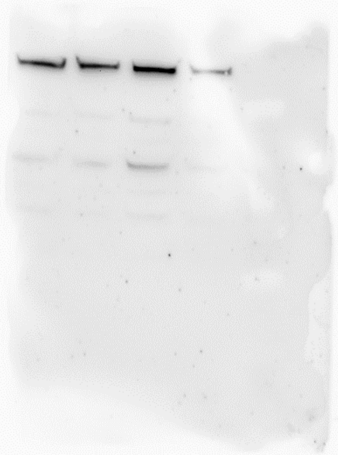

C

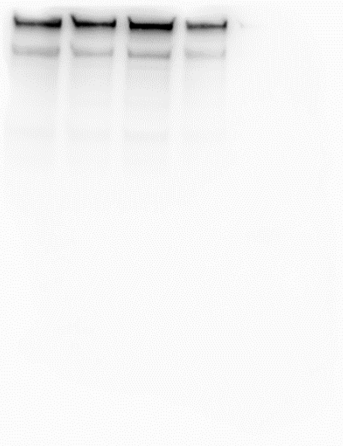

Supplementary figure S9: Full size Western blot figures for A = GAPDH, B = NPHS1 and C = SYNPO.
Samples were loaded from left to right: UM48 +RA, UM48 -RA, UF31 +RA and UF31 -RA.

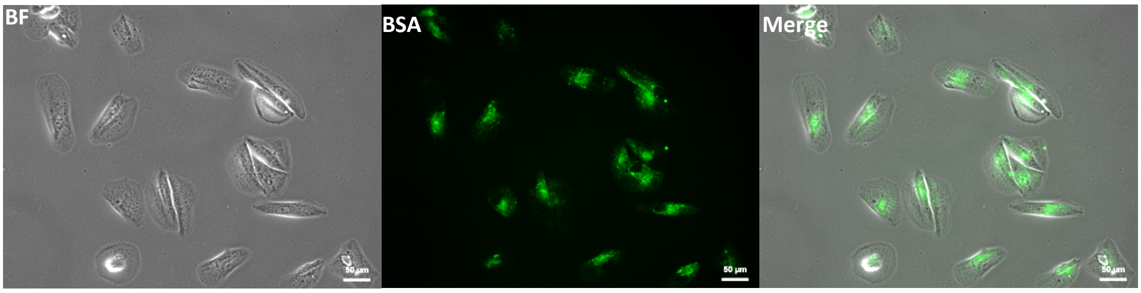

Supplementary figure S10: Albumin uptake assay in UM51 podocytes. Infiltration of exogenous BSA supplemented into the culture medium confirmed endocytosis of Albumin in UM51 podocytes.

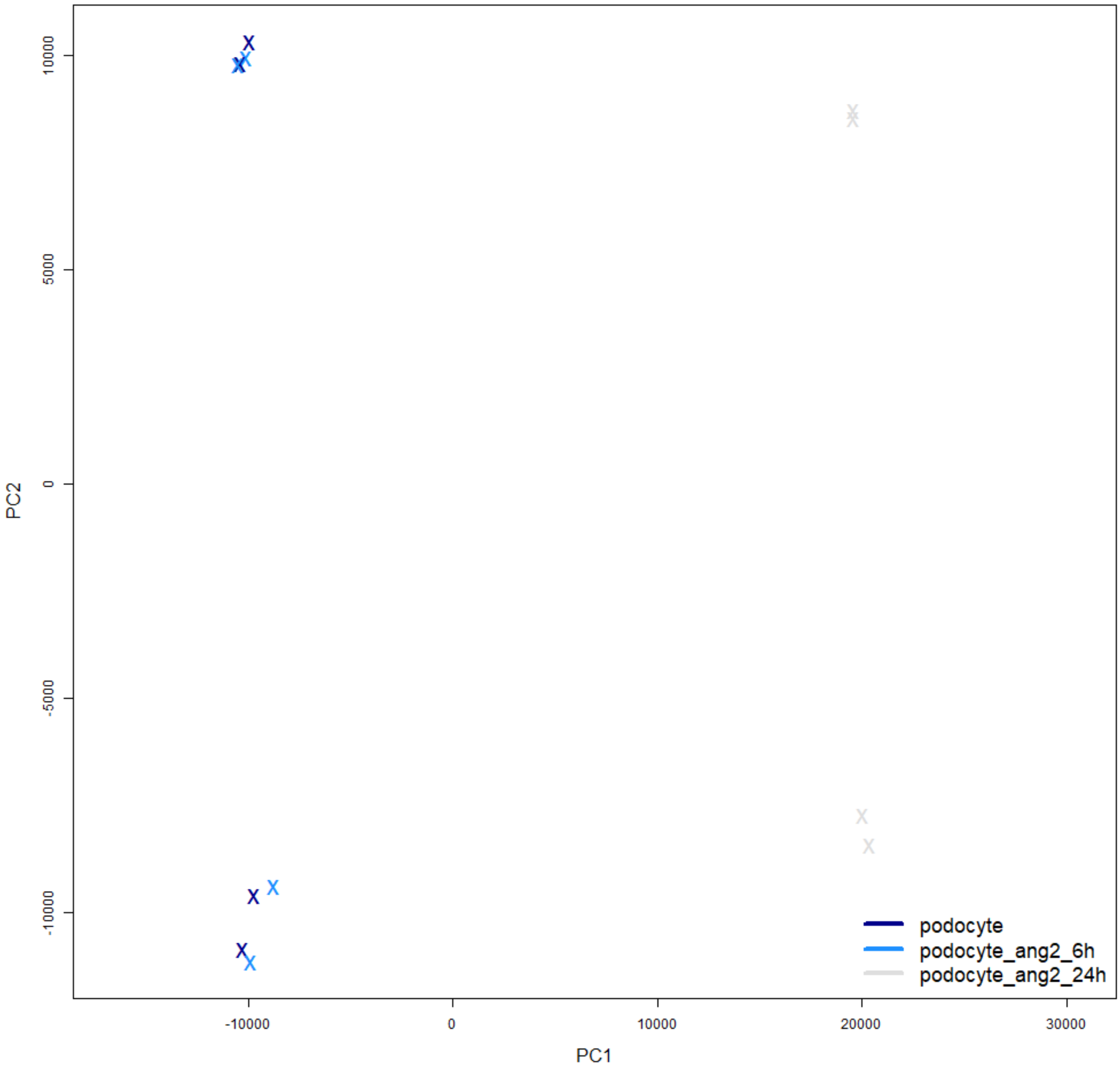

Supplementary figure S11: Principal component analysis of podocytes UM48 and UM51 with and without 6h and 24h of ANGII treatment. For the Principal component analysis (PCA) expression data was filtered for a coefficient of variation greater than 0.3. The PCA was made with the R Built-in function *prcomp* and two principal components were plotted.
